# Redefining Parameter Estimation and Covariate Selection via Variational Autoencoders: One Run Is All You Need

**DOI:** 10.1002/psp4.70129

**Published:** 2025-11-04

**Authors:** Jan Rohleff, Freya Bachmann, Uri Nahum, Dominic Bräm, Britta Steffens, Marc Pfister, Gilbert Koch, Johannes Schropp

**Affiliations:** ^1^ Department of Mathematics and Statistics University of Konstanz Konstanz Germany; ^2^ Pediatric Pharmacology and Pharmacometrics, University Children's Hospital Basel (UKBB) University of Basel Basel Switzerland; ^3^ Institute of Biomedical Engineering and Medical Informatics University of Applied Sciences and Arts Northwestern Switzerland (FHNW) Olten Switzerland; ^4^ School of Life Sciences University of Applied Sciences and Arts Northwestern Switzerland (FHNW) Olten Switzerland

**Keywords:** Bayesian inference, covariate selection, data‐driven modeling, generative artificial intelligence (AI), machine learning, nonlinear mixed effects modeling, parameter estimation, variational autoencoder

## Abstract

Generative Artificial Intelligence (AI) frameworks, such as Variational Autoencoders (VAEs), have proven powerful in learning structured representations from complex, high‐dimensional data. In pharmacometrics (PMX), nonlinear mixed effects (NLME) modeling is widely used to capture inter‐individual variability and link covariates to characterize parameters with the goal of informing key decisions in drug research and development. This research combines the strengths of both approaches by introducing a VAE framework specifically designed for NLME modeling. The proposed method integrates the flexibility of generative AI with the interpretability and robustness of mechanism‐based PMX modeling. To advance covariate selection in PMX, we replace the Evidence Lower Bound objective in VAEs with an objective function based on the corrected Bayesian information criterion. This enables the simultaneous evaluation of all potential covariate‐parameter combinations, thereby allowing for automated and joint estimation of population parameters and covariate selection within a single run. Manual selection and repeated model fitting across covariate combinations are no longer required. We demonstrate the effectiveness of this combined AI‐PMX approach with two representative cases. As the first generative AI‐based optimization method for NLME modeling, the VAE achieves high‐quality results in a single run, outperforming traditional stepwise procedures in terms of efficiency. As such, the presented approach facilitates automated model development, advancing PMX and its applications in model‐informed drug development.

**Study Highlights**

- What is the current knowledge on the topic?
  ○ Variational Autoencoders (VAEs) have been explored in pharmacometrics modeling. However, they have not yet been adapted to nonlinear mixed effects (NLME) frameworks or applied to covariate selection.
- What question did this study address?
  ○ Can we develop a robust, efficient, and flexible AI framework for NLME modeling using VAEs?
  ○ How competitive are VAEs compared to the established methods in estimating population parameters and selecting covariates?
  ○ Do they offer advantages in computational efficiency and automated covariate selection?
- What does this study add to our knowledge?
  ○ VAEs are a powerful AI framework for efficiently solving NLME modeling problems and opening new opportunities in pharmacometrics.
  ○ They estimate population parameters and select covariates simultaneously in one single run, making the modeling process less time‐consuming while maintaining the quality of results.
- How might this change drug discovery, development, and/or therapeutics?
  ○ VAEs adapted to NLME frameworks represent a significant advancement in the field of PMX. Combining AI and PMX modeling can lead to more accurate and more efficient automated model assessment and building.

## 1 Introduction

Model‐informed drug development (MIDD) leverages pharmacometrics (PMX) modeling and simulation to support key decisions in drug research, development, and regulatory review [1]. Nonlinear mixed effects (NLME) modeling characterizes PMX mechanisms by accounting for fixed effects (population parameters) and random effects (inter‐individual variability) through the maximization of the associated log‐likelihood function [2], which cannot be computed in closed form. Classical solution methods in NLME modeling include linearization‐based approaches such as First‐Order Conditional Estimation (FOCE) [3] and stochastic approximation maximization techniques such as the SAEM algorithm [4].

PMX plays a central role in MIDD, but model development remains a cumbersome and slow process due to complex model structures and often high computational demands. In particular, covariate selection is hard to automate and often results in suboptimal models.

In PMX, NLME modeling generally follows a sequential process [5]. First, the structural model is developed and the corresponding model parameters, that is, fixed and random effects, are estimated. Second, covariate selection is performed, which is often the most time‐consuming part. Current automated covariate selection methods, such as SCM [6], SAMBA [7] and COSSAC [8], rely on iterative testing of potential covariate‐parameter relationships. Each iteration requires estimating model parameters and potential covariate effects by maximizing the log‐likelihood. Covariate selection is then based on information criteria such as the Akaike Information Criterion (AIC) [9], the Bayesian Information Criterion (BIC) [10], or the corrected BIC (BICc) [11].

NLME modeling can be viewed as a Bayesian inference problem [12, 13], since individual parameters are assumed to follow a population distribution (the prior), and the observed data update our knowledge about these parameters (the posterior). Variational autoencoders (VAEs) [14] are generative artificial intelligence (AI) methods that combine neural networks with Bayesian principles to estimate unobservable variables from the data. These properties make VAEs a powerful and flexible tool for solving complex Bayesian inference problems, such as those in NLME modeling. Initial applications of VAEs in PMX have been presented for PK modeling [15, 16, 17].

In this paper, we present an adapted VAE for NLME modeling. First, the VAE is configured to reflect the structure of NLME frameworks, incorporating a population prior and structuring the latent space around individual model parameters. Second, the VAE is augmented to perform automated covariate selection. This is achieved by estimating a full covariate effect matrix, enabling the evaluation of all possible covariate–parameter combinations in a single step. Delattre et al. [11] proposed using the BICc for covariate selection, as it properly accounts for the random effects structure. To evaluate all possible covariate–parameter combinations at once, our adapted VAE employs a BICc‐based criterion as the loss function, enabling efficient covariate selection in one run.

In conclusion, our adapted VAE simultaneously estimates model parameters, that is, fixed and random effects, and performs automated covariate selection within the NLME framework. The proposed combined AI‐PMX approach is demonstrated using two case studies, the well‐known theophylline dataset [18], and a more complex neonatal weight progression dataset [19, 20]. The presented VAE, implemented in Python, represents the first generative AI‐based optimization method for NLME modeling capable of completing both model parameter estimation and covariate selection in one single run.

## 2 Methods

This Section first provides the theoretical basis of the NLME framework (Section 2.1), followed by a description of how VAEs can be integrated within NLME frameworks (Section 2.2). Finally, we present our augmented VAE approach designed for automated covariate selection (Section 2.3).

### 2.1 Nonlinear Mixed Effects Model

Consider a dataset of N individuals, where the data for each individual i is given by

$$D_{i}=\left(t_{\mathrm{ij}},x_{\mathrm{ij}},c_{i};1\leq j\leq n_{i}\right)$$

for 1≤i≤N. Here, $t_{\mathrm{ij}}$ represents the time of the j‐th observation $x_{\mathrm{ij}}\in \mathbb{R}$, with $n_{i}\in \mathbb{N}$ denoting the total number of observations for individual i. The vector $c_{i}\in {\mathbb{R}}^{n_{c}}$ contains $n_{c}\in \mathbb{N}$ individual covariate (relations). Furthermore, $n_{z}\in \mathbb{N}$ denote the number of individual parameters applied in the model. For each individual i, the structural model reads

(1) $$\frac{d}{dt}y_{i}\left(t\right)=f\left(t,y_{i}\left(t\right),\zeta_{i}\right)\;\text{for}\;t\in \left(0,T\right],\;y_{i}\left(0\right)=y_{0}\left(\zeta_{i}\right),$$

where f represents the mechanism of the model. The error model satisfies

(2) $$x_{ij}=g\left(t_{ij},y_{i}\left(t_{ij}\right),\zeta_{i}\right)+\epsilon_{ij}\;\text{for}\;j=1,\ldots ,n_{i},$$

with an output function g. The residual errors are assumed to follow a normal distribution with variance $a^{2}$, that is, $\epsilon_{\mathrm{ij}}∼N\left(0,a^{2}\right)$. For the sake of readability, we choose a constant error model, however, the framework is flexible and allows any error model specification. The variable $\zeta_{i}$ is given by the individual model (prior distribution)

(3) $$\begin{array}{c} \begin{array}{ll} h\left(\zeta_{i}\right)=z_{i} & \text{for}\;i=1,\ldots ,N,\\ z_{i}=z_{\mathrm{pop}}+\beta c_{i}+\eta_{i},\;\eta_{i}∼N\left(0,\Omega \right), & \Omega =\mathrm{diag}\left(\omega_{1}^{2},\ldots ,\omega_{n_{z}}^{2}\right), \end{array} \end{array}$$

where $z_{\mathrm{pop}}\in {\mathbb{R}}^{n_{z}}$ denotes the fixed effects, $\beta =\left(\beta_{1},\ldots ,\beta_{n_{c}}\right)$ $\in {\mathbb{R}}^{n_{z}\times n_{c}}$ is a matrix of possible covariate effect parameters, and h denotes a smooth invertible transformation to adjust the prior to more general non‐Gaussian distributions. The population parameters of the model Equations ((1), (2), (3)) are given by $\theta =\left(z_{\mathrm{pop}},\beta ,\Omega ,a\right).$ The likelihood function of the observations $x=\left(x_{\mathrm{ij}};1\leq i\leq N,1\leq j\leq n_{i}\right)$ is expressed as

$$p\left(x\right)=p\left(x;\theta \right)=\int p\left(x,z\right)\mathrm{dz}=\int p\left(x|z\right)p\left(z\right)\mathrm{dz}.$$

We denote the corresponding log‐likelihood by

$$\mathrm{LL}\left(\theta \right)=\mathrm{LL}\left(x;\theta \right)=\log p\left(x;\theta \right).$$

The objective is to maximize the log‐likelihood function $\mathrm{LL}\left(\theta \right)$ with respect to the population parameters θ, that is,

(4) $$\max_{\theta }\mathrm{LL}\left(\theta \right)$$

referred to as marginalized likelihood problem. A commonly used solution for this problem is the SAEM algorithm [4] available in Monolix 2024 [21]. In 2013, Kingma and Welling introduced in VAEs [14, 22] as a powerful AI‐based alternative that offers an efficient way to approximate and optimize marginalized likelihoods. This motivates our application of VAEs in NLME modeling to solve Equation (4).

### 2.2 VAEs for NLME Modeling

**FIGURE 1:**
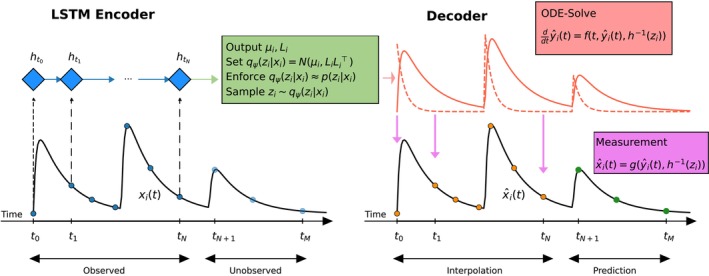
The Variational Autoencoder (VAE) framework. In the left panel, the LSTM encoder infers the latent variable $z_{i}$ from the measurements $x_{i}$. The encoder posterior distribution $q_{\psi }\left(z_{i}|x_{i}\right)$ is enforced to match the true posterior distribution $p\left(z_{i}|x_{i}\right)$. In the right panel, the decoder reconstructs the measurements ${\hat{x}}_{i}$ from the latent variable $z_{i}$.

In this subsection, we design a VAE to solve marginalized likelihood problems Equation (4) specifically adapted to the structure of NLME frameworks. The VAE architecture consists of two main components, encoder and decoder. The encoder infers the latent variables $z_{i}$ from the observed data $x_{i}=\left(x_{\mathrm{ij}};1\leq j\leq n_{i}\right)$, while the decoder reconstructs the data based on the latent representation. Training is performed by maximizing the Evidence Lower Bound (ELBO) of the marginal log‐likelihood $\mathrm{LL}\left(\theta \right)$. An overview of the VAE architecture is illustrated in Figure 1.

#### 2.2.1 Encoder

The objective of the encoder is to propose a smooth, parametrized approximation $q_{\psi }\left(z_{i}|x_{i}\right)$ of the posterior distribution $p\left(z_{i}|x_{i}\right)$, which extracts the individual model parameters $z_{i}$ from the observations $x_{i}$ for every individual i. The distribution $q_{\psi }\left(z_{i}|x_{i}\right)$ is modeled using a Long Short‐Term Memory (LSTM) neural network architecture (see [23], Ch. 10.10) with subject‐independent network parameters ψ

(5)

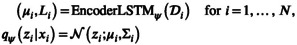

with $\sum_{i}=L_{i}L_{i}^{⊤}$, where $L_{i}$ a lower‐triangular matrix with positive diagonal entries. The approximated posterior distribution $q_{\psi }\left(z_{i}|x_{i}\right)=N\left(z_{i};\mu_{i},\sum_{i}\right)$ is utilized to sample the latent variable $z_{i}\in {\mathbb{R}}^{n_{z}},$ that is,

(6) $$z_{i}=\mu_{i}+L_{i}\varepsilon_{i},\;\varepsilon_{i}∼N\left(0,I\right),$$

or equivalently, $z_{i}∼q_{\psi }\left(z_{i}|x_{i}\right).$

#### 2.2.2 Decoder

The decoder utilizes the sampled latent variable $z_{i}$ in the structural model Equation (1) to generate the model prediction ${\hat{y}}_{i}\left(t\right)$. From this, the predicted observation ${\hat{x}}_{i}$ is reconstructed according to the error model Equation (2).

#### 2.2.3 Loss Function and Training

An ELBO loss function, adapted to the NLME framework, is derived to enable VAE training. The error in the measurements is incorporated into the ELBO loss function using the log‐likelihood, assuming $x_{\mathrm{ij}}|z_{i}∼N\left({\hat{x}}_{\mathrm{ij}},a^{2}\right).$ In case of a one‐dimensional observation, this leads to

$$\begin{array}{c} \log p\left(x|z\right)=\sum_{i=1}^{N}\log p\left(x_{i}|z_{i}\right)=-\frac{N_{\mathrm{tot}}}{2}\left(\log\left(2\pi \right)+\log\left(a^{2}\right)\right)\\ \;-\frac{1}{2}\sum_{i=1}^{N}\sum_{j=1}^{n_{i}}{\left(\frac{x_{\mathrm{ij}}-{\hat{x}}_{\mathrm{ij}}\left(z_{i}\right)}{a}\right)}^{2} \end{array}$$

with $N_{\mathrm{tot}}=\sum_{i=1}^{N}n_{i}$. To ensure that the sampled latent variables $z_{i}$ (see Equation (6)) fit into the population, that is,

(7) $$z_{i}=z_{\mathrm{pop}}+\beta c_{i}+\eta_{i},\;\eta_{i}∼N\left(0,\Omega \right),$$

the ELBO loss function includes a Kullback–Leibler divergence term (see [23], Ch. 3.13)

$$D_{\mathrm{KL}}\left(q_{\psi }\left(z_{i}|x_{i}\right)\;\|\;p\left(z_{i}\right)\right)=D_{\mathrm{KL}}\left(N\left(\mu_{i},\sum_{i}\right)\|\;N\left(z_{\mathrm{pop}}+\beta c_{i},\Omega \right)\right).$$

Hence, the ELBO loss function with the population parameter $\theta =\left(z_{\mathrm{pop}},\beta ,\Omega ,a\right)$ for the marginalized likelihood problem Equation (4) reads

(8) $$L_{\psi }\left(x;\theta \right)=\sum_{i=1}^{N}E_{z_{i}∼q_{\psi }\left(\cdot |x_{i}\right)}\left[\log p\left(x_{i}|z_{i}\right)\right]-\sum_{i=1}^{N}D_{\mathrm{KL}}\left(q_{\psi }\left(z_{i}|x_{i}\right)\;\|\;p\left(z_{i}\right)\right).$$

The ELBO loss function is a lower bound of the log‐likelihood function, that is, $L_{\psi }\left(x;\theta \right)\leq \mathrm{LL}\left(x\right)$. More precisely, the relation

(9) $$L_{\psi }\left(x;\theta \right)=\mathrm{LL}\left(x;\theta \right)-D_{\mathrm{KL}}\left(q_{\psi }\left(z|x\right)\;\|\;p\left(z|x\right)\right)$$

holds, where $p\left(z|x\right)=\prod_{i=1}^{N}p\left(z_{i}|x_{i}\right)$ and $q_{\psi }\left(z|x\right)=\prod_{i=1}^{N}q_{\psi }\left(z_{i}|x_{i}\right)$. Hence, the marginalized likelihood problem Equation (4) can be solved by maximizing the ELBO loss function in Equation (8) with respect to the population parameters $\theta =\left(z_{\mathrm{pop}},\beta ,\Omega ,a\right)$ and the network parameters ψ. Population parameters $\theta_{k}$ are updated done according to the global uniquely solvable maximization step

(10) $$\theta^{\left(k+1\right)}=\arg\max_{\theta }L_{\psi^{\left(k\right)}}\left(x;\theta \right).$$

#### 2.2.4 Implementation Details

VAEs are potentially sensitive to architectural hyperparameters (e.g., number of layers, hidden dimension) and parameter initialization, which can affect performance and stability. They can also get trapped in undesirable local minima of Equation (4) during training [14]. Thus, inspired by the SAEM algorithm, we incorporate a burn‐in phase consisting of a priori NLME investigations and Kullback–Leibler annealing [24], effectively pre‐training the LSTM encoder. This enhances stability and enables the VAE to escape undesired local minima.

#### 2.2.5 Accuracy and Prediction Quality of Solutions

The quality of the VAE solutions with respect to the likelihood function depends on the size of the gap between the ELBO loss function $L_{\psi }\left(x;\theta \right)$ and the log‐likelihood $\mathrm{LL}\left(\theta \right)$, which is called the tightness of the bound. According to Equation (9), this gap corresponds to the Kullback–Leibler divergence term between $q_{\psi }\left(z|x\right)$ and $p\left(z|x\right)$. This means, the better the encoder distribution $q_{\psi }\left(z|x\right)$ approximates the true posterior $p\left(z|x\right)$, the smaller the gap and the better the likelihood value of the VAE solutions.

Classical methods, such as FOCE, linearize the model by using a first‐order Taylor expansion, which results in Gaussian posteriors. However, SAEM‐based methods use Markov Chain Monte Carlo (MCMC) iterations to approximate the posterior. MCMC‐based approximations are quite accurate, but computationally costly, especially for large populations. In contrast to classical methods, the VAE derives the encoder distribution $q_{\psi }\left(z|x\right)$ directly from the data, using a LSTM neural network smoothly parametrized by its network parameters ψ. Since the network parameters ψ are subject‐independent, the VAE potentially improves data structure readout and prediction quality. Using a trained encoder $q_{\psi }\left(z|x\right)$, one can infer the full posterior distribution of the latent variables z, as well as perform maximum a posteriori (MAP) estimations, both by simple evaluations of the LSTM neural network without calculating any integrals or solving optimization problems.

### 2.3 Augmented VAEs for Covariate Selection

Based on the VAE concept, we propose a novel approach for covariate selection. To assess the quality of a covariate model, it is not sufficient to maximize the log‐likelihood $\mathrm{LL}\left(\theta \right)$ alone. Instead, a model selection criterion that balances goodness‐of‐fit and model complexity is required. For this purpose, we aim to minimize the BICc [11].

(11) $$\text{BICc}=-2\mathrm{LL}\left(\theta \right)+\log\left(N\right)\cdot \dim\left(\theta_{R}\right)+\log\left(N_{\mathrm{tot}}\right)\cdot \dim\left(\theta_{F}\right),$$

where $\dim\left(\theta_{R}\right)$ and $\dim\left(\theta_{F}\right)$ denote the number of the random and fixed effects, respectively.

Given covariate vectors $c_{i}$ for individuals i=1,…,N, the augmented VAE estimates the full covariate effect matrix

$$\beta =\left(\beta_{1}\;\cdots \;\beta_{n_{c}}\right)\in {\mathbb{R}}^{n_{z}\times n_{c}},$$

where each column $\beta_{j}\in {\mathbb{R}}^{n_{z}}$, for $j\in \left\{1,\ldots ,n_{c}\right\}$, describes how the j‐th covariate affects the model parameters. If an entire column $\beta_{j}$ is estimated to be zero, the corresponding j‐th covariate is deemed irrelevant and excluded from the final model. Conversely, if any entry in the column is non‐zero, that is, $\beta_{\mathrm{jk}}\neq 0$ for some $k\in \left\{1,\ldots ,n_{z}\right\}$, the j‐th covariate is expected to have an effect on the parameter $z_{k}$. Consequently, estimating the covariate effect matrix β corresponds to selecting the most appropriate combination of all possible covariate‐parameter combinations.

#### 2.3.1 BICc‐ELBO Loss Function

For the augmented VAE, we introduce the BICc‐ELBO loss function that incorporates the appropriate penalty term for any possible covariate‐parameter combination. Based on the structure of the BICc function (compare Equation (11)) the BICc‐ELBO loss function reads

(12) $$L_{\psi }^{\text{BICc}}\left(x,\theta \right)=-2L_{\psi }\left(x;\theta \right)+\log\left(N\right){\left\|\beta \right\|}_{0}+C$$

with C being a constant that does not affect the optimization. In Equation (12), the term ${\left\|\beta \right\|}_{0}$ denotes the $L_{0}$‐(pseudo) norm of β, that is, the number of non‐zero elements in the covariate effect matrix β, which coincides with the number of relevant covariate effects. Minimizing the BICc‐ELBO loss function Equation (12) selects the best covariate combination with respect to BICc from all covariate‐parameter combinations.

The BICc‐ELBO is an upper bound for BICc (BICc ≤ $L_{\psi }^{\text{BICc}}\left(x,\theta \right)$), so we can optimize the BICc by minimizing the BICc‐ELBO. Minimizing the BICc‐ELBO loss function Equation (12) is similar to maximizing the ELBO loss function Equation (8), with a different update of the fixed effects $\left(z_{\mathrm{pop}},\beta \right),$ while updates for Ω and a remain equivalent. For the ELBO loss function, the parameters $\theta =\left(z_{\mathrm{pop}},\beta ,\Omega ,a\right)$ are updated according to Equation (10). In contrast, the BICc‐ELBO loss function requires solving the optimization problem
(13)

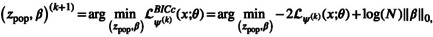

which is NP‐hard [25]. However, the quadratic structure of $L_{\psi }$ in $\left(z_{\mathrm{pop}},\beta \right)$ allows to efficiently compute global solutions of (13) using Mixed Integer Quadratic Programming (MIQP), even with hundreds of covariates [26]. Update Equation (13) allows to simultaneously estimate the relevant covariate effects (i.e., determine β) and the population parameters θ. Since the optimization problems defined in Equations (10) and (13) admit unique global solutions, the stability and convergence properties of the VAE are preserved when replacing the loss function $L_{\psi }$ with $L_{\psi }^{\text{BICc}}$.

## 3 Results

Two real‐world case studies are applied to assess the performance of the proposed VAE approach. The first case study focuses on the pharmacokinetics of theophylline [18]. The second investigates the weight progression of neonates during the first 7 days of life [19, 20].

For each of the two case studies, we describe the analysis dataset, the applied model, and the numerical results. For parameter estimation, we compare the VAE results with those obtained using the SAEM algorithm [4] for fixed covariates. Additionally, we benchmark our automated covariate selection against the SAEM‐based methods SAMBA, COSSAC, and SCM using the BICc criterion computed via importance sampling.

Implementation is done in Python 3.13 using PyTorch [27], utilizing the package TorchODE [28] for parallelized ordinary differential equation (ODE) solving. The VAE employs a single LSTM layer, with the hidden dimensions depending on the number of individuals and model parameters. It is trained using the Adam optimizer [29], which automatically adapts the learning rate for each parameter. For reproducibility, the random seed was fixed. All learning parameters are provided in the Supporting Information S1. Computations were performed on a MacBook Pro equipped with an Apple M4 Pro chip (12‐core CPU, 24 GB RAM). VAE examples are available on GitHub (https://github.com/janrohleff/vae_nlme).

### 3.1 Case Study 1—Theophylline Pharmacokinetics

#### 3.1.1 Data

The dataset consists of theophylline concentration measurements in the blood plasma of N=12 patients. All patients received a theophylline loading dose at time t=0. Concentration measurements $C_{i}\left(t_{\mathrm{ij}}\right)$ were taken at various time points $t_{\mathrm{ij}}$ for each individual i=1,…,N and measurement $j=1,\ldots ,n_{i}$. Additionally, each individual's baseline weight $w_{i}$ and sex $\mathrm{se}x_{i}$ were recorded as covariates. The available data of individual i is represented by

$$D_{ij}=\left(\begin{array}{c} t_{ij}\\ C_{i}\left(t_{ij}\right)\\ w_{i}\\ sex_{i} \end{array}\right)\in {\mathbb{R}}^{4}\;\text{for}\;i=1,\ldots ,N\;\text{and}\;j=1,\ldots ,n_{i}.$$

#### 3.1.2 Model Description

Theophylline concentrations are characterized by a one‐compartment model with first‐order absorption and elimination [18] given by
(14)

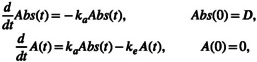

where $\mathrm{Abs}\left(t\right)$ and $A\left(t\right)$ denote the amount of theophylline in the absorption and central compartment. The parameters $k_{a}$ and $k_{e}$ are the absorption and elimination rate constants. D is the amount of drug given to the individual at time t=0. The observed concentration in the central compartment, given by $g\left(A\left(t\right),V\right)=A\left(t\right)/V$, is

$$C\left(t\right)=\frac{A\left(t\right)}{V}.$$

We assume that the parameters $\left(k_{a,i},k_{e,i},V_{i}\right)\in {\mathbb{R}}^{3}$ follow a log‐normal distribution, that is, $h\left(x\right)=\log\left(x\right)$ and

$$\begin{array}{ll} \log\left(k_{a,i}\right)=\log\left(k_{a,\mathrm{pop}}\right)+\beta_{k_{a}}c_{i}+\eta_{k_{a},i}, & \eta_{k_{a},i}∼N\left(0,\omega_{k_{a}}^{2}\right),\\ \log\left(k_{e,i}\right)=\log\left(k_{e,\mathrm{pop}}\right)+\beta_{k_{e}}c_{i}+\eta_{k_{e},i}, & \eta_{k_{e},i}∼N\left(0,\omega_{k_{e}}^{2}\right),\\ \log\left(V_{i}\right)=\log\left(V_{\mathrm{pop}}\right)+\beta_{V}c_{i}+\eta_{V,i}, & \eta_{V,i}∼N\left(0,\omega_{V}^{2}\right). \end{array}$$

Note that any invertible transformation h, as shown in Equation (3), can be chosen to model alternative parameter distributions. The covariate $c_{i}=\left(c_{i}^{w},c_{i}^{\mathrm{sex}}\right)\in {\mathbb{R}}^{2}$ is defined as

$$c_{i}^{w}=\log\left(\frac{w_{i}}{w_{\mathrm{pop}}}\right)\;\;\text{and}\;\;c_{i}^{\mathrm{sex}}=\left\{\begin{array}{cc} 1 & \text{for individual}\;i\;\text{is}\;a\;\text{male}\\ 0 & \;\;\;\;\text{for individual}\;i\;\text{is}\;a\;\text{female} \end{array}\right.,$$

and $w_{\mathrm{pop}}$ denotes the mean weight over the N individuals. The full covariate effect matrix reads

$$\beta =\left(\begin{array}{c} \beta_{k_{a}}\\ \beta_{k_{e}}\\ \beta_{V} \end{array}\right)=\left(\begin{array}{cc} \beta_{k_{a}}^{w} & \beta_{k_{a}}^{\mathrm{sex}}\\ \beta_{k_{e}}^{w} & \beta_{k_{e}}^{\mathrm{sex}}\\ \beta_{V}^{w} & \beta_{V}^{\mathrm{sex}} \end{array}\right)\in {\mathbb{R}}^{3\times 2}.$$

#### 3.1.3 Numerical Results

**FIGURE 2:**
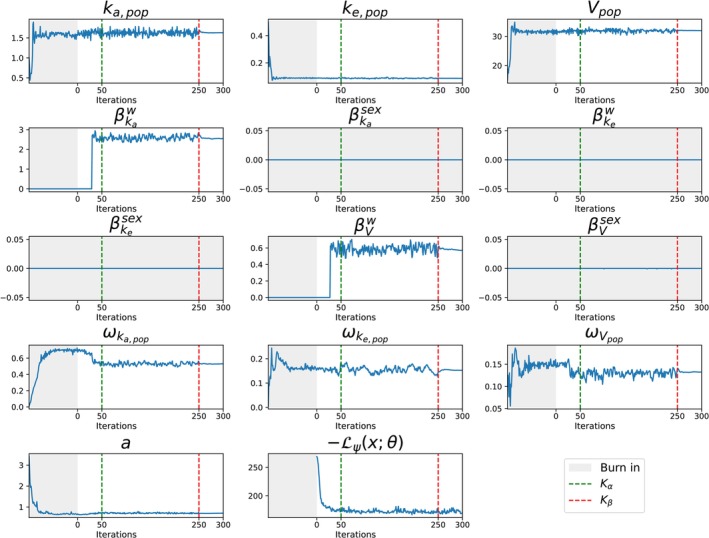
Theophylline example. Convergence of the population parameters θ (including covariate effects β) for the theophylline data set. The VAE starts with a burn‐in phase of 100‐iteration (gray area), followed by the main training phase. The dashed green line marks the point where the Kullback–Leibler divergence term is fully annealed; the dashed red line marks the smoothing phase of the maximization step. Covariate effects excluded from the final model (i.e., converging to zero) are shown on a light gray background.

Running the VAE results in the final optimal covariate effect matrix $\beta^{*}$ with non‐zero covariate effects $\beta_{V}^{w}$, $\beta_{k_{a}}^{w},$ and all other covariate effects set to zero (see Figure 2). The VAE converges in up to 250 iterations. It is important to note that the VAE selection process follows the standard selection rules. In particular, the addition of a covariate effect at a given iteration is accompanied by a simultaneous reduction in the corresponding variance. At iteration 30, the uptake of $\beta_{V}^{w}$ and $\beta_{k_{a}}^{w}$ coincides with a decrease in $\omega_{V}$ and $\omega_{k_{a}}$, respectively (see Figure 2). Detailed results are summarized in Table 1. In this example, the estimated population parameters and log‐likelihood values are similar across all methods, apart from some stochastic noise. The run is repeated in the Supporting Information S2 with different initial seeds and varying hidden dimensions in the LSTM layer. The results show that the VAE consistently computes the same solution up to negligible differences.

**TABLE 1:** Theophylline example.

	VAE		COSSAC		SAMBA		SCM	
**Fixed effects**								
$k_{a,\mathrm{pop}}$	1.63		1.60		1.64		1.60	
$k_{e,\mathrm{pop}}$	0.086		0.087		0.084		0.087	
$V_{\mathrm{pop}}$	31.97		31.82		32.21		31.82	
**Covariates**								
$\beta_{k_{a}}^{w}$	2.55		2.58		2.53		2.58	
$\beta_{k_{a}}^{\mathrm{sex}}$	—		—		—		—	
$\beta_{k_{e}}^{w}$	—		—		—		—	
$\beta_{k_{e}}^{\mathrm{sex}}$	—		—		—		—	
$\beta_{V}^{w}$	0.57		0.57		—		0.57	
$\beta_{V}^{\mathrm{sex}}$	—		—		—		—	
**Standard deviation**								
$\omega_{k_{a}}$	0.53		0.54		0.55		0.54	
$\omega_{k_{e}}$	0.15		0.15		0.15		0.15	
$\omega_{V}$	0.13		0.13		0.14		0.13	
**Error model**								
a	0.71		0.73		0.73		0.73	
**Stat. criteria**								
	**Lin**	**IS**	**Lin**	**IS**	**Lin**	**IS**	**Lin**	**IS**
−2LL	331.1	332.2	330.4	331.9	333.4	335.1	330.4	331.9
BICc	362.7	363.8	362.0	363.5	362.5	364.2	362.0	363.5
Runs	1		3		2		16	

*Note:* Comparison of model parameters estimated by VAE, COSSAC, SAMBA and SCM. The likelihood function −2LL and the BICc criterion are computed by a linearization method (Lin) and by importance sampling (IS). The number of runs is given in the last row.

Looking at the covariate model, COSSAC and SCM select the same optimal covariate effect matrix $\beta^{*}$ as the VAE, whereas SAMBA suggests only the covariate effect $\beta_{V}^{w}$. The BICc criterion indicates that the covariate model selected by the VAE, COSSAC and SCM is slightly better in terms of model quality.

The VAE stands out in terms of efficiency, requiring only one single run to simultaneously estimate population model parameters and select covariates. SAMBA and COSSAC also perform well but require multiple runs (two for SAMBA and three for COSSAC), each involving optimization and computing of the log‐likelihood function for a fixed covariate model. SCM is the slowest, requiring 16 runs to reach similar results.

Some remarks: (i) In this example, a VAE population fit with fixed covariates, that is, estimating population parameters only, takes 5.8 s. If we include covariate selection, the total time is 6.2 s. This means, for this small example, covariate selection only adds about 6% CPU time. (ii) Multiple dosing can be handled by the VAE. We tested the same model under a multiple dosing regimen, administering 10 doses every 10 h. The results are consistent with those obtained from the single‐dose setting. The corresponding experiment and implementation details are available on GitHub.

### 3.2 Case Study 2—Neonatal Weight Progression

#### 3.2.1 Data

The aim was to develop a model to characterize weight progression over the first 7 days of life using data from N=2425 neonates [20]. Each neonate's weight $W_{i}\left(t_{\mathrm{ij}}\right)$ was measured at various time points $t_{\mathrm{ij}}$, where i=1,…,N and $j=1,\ldots ,n_{i}$. The clinically relevant covariates include sex $\left(\mathrm{sex}\right)$, mode of delivery $\left(\text{DelM}\right)$, gestational age $\left(\mathrm{GA}\right)$, maternal age $\left(\text{Mage}\right)$, and parity $\left({\text{Para}}_{2}\right)$. The available data is represented as

$$D_{ij}=\left(\begin{array}{c} t_{ij}\\ W_{i}\left(t_{ij}\right)\\ sex_{i}\\ DelM_{i}\\ GA_{i}\\ Mage_{i}\\ Para_{2i} \end{array}\right)\in {\mathbb{R}}^{7}\;\text{for}\;i=1,\ldots ,N\;\text{and}\;j=1,\ldots ,n_{i}.$$

**FIGURE 3:**
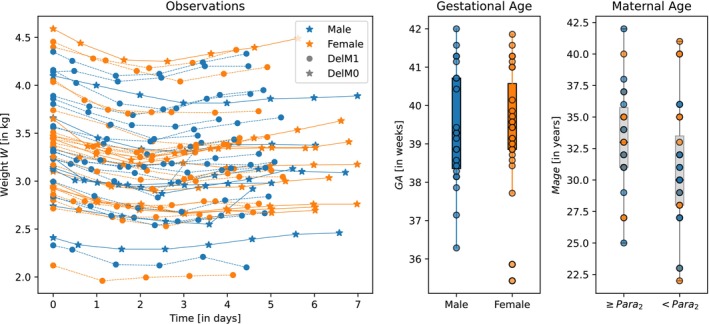
Neonatal weight progression example. The neonate weight data set with clinical covariates sex (*sex*), mode of delivery (*DelM*), gestational age (*GA*), maternal age (*Mage*), and parity (*Para*_2_).

The dataset for the first 50 neonates is visualized in Figure 3.

#### 3.2.2 Model Description

We consider a slightly updated version of the structural model from [20], in which the original discontinuity in the lag time has been smoothed out, which is more realistic from a physiological perspective and ensures differentiability. This modification enables gradient‐based training of the VAE's encoder. Maturation effects are incorporated with postnatal age‐dependent zero‐order production and first‐order elimination terms. The structural model reads

(15) $$\frac{d}{\mathrm{dt}}W\left(t\right)=k_{\text{prod}}\left(t\right)-k_{\mathrm{el}}\left(t\right)W\left(t\right),\;W\left(0\right)=W_{0},$$

where

$$k_{\text{prod}}\left(t\right)=\frac{k_{\text{in}}}{1+\exp\left(-2\left(t-T_{\mathrm{lag}}\right)\right)}\;\text{and}\;k_{\mathrm{el}}\left(t\right)=k_{\text{out}}\left(1-\frac{t}{T_{50}+t}\right).$$

The production term $k_{\text{prod}}\left(t\right)$ is modeled by a modified sigmoid function and can be interpreted as a smooth delay of $T_{\mathrm{lag}}$ days. The structural model contains five parameters $\left(W_{0},k_{\text{in}},T_{\mathrm{lag}},k_{\text{out}},T_{50}\right)\in {\mathbb{R}}^{5}$, each with inter‐individual variability and assumed to follow a log‐normal distribution. Covariates *sex*, *DelM*, *Para*_2_ are categorical, and *GA* and *Mage* are continuous and centered around their mean value as in the previous example, that is, $c_{i}^{\text{Mage}}=\text{log}\left({\text{Mage}}_{i}/{\text{Mage}}_{\mathrm{pop}}\right)$ and $c_{i}^{\mathrm{GA}}=\log\left({\mathrm{GA}}_{i}/{\mathrm{GA}}_{\mathrm{pop}}\right)$. Five parameters with five covariates result in $2^{25}$ possible covariate‐parameter combinations.

#### 3.2.3 Numerical Results

First, we perform a population fit of model Equation (15) without including any covariates to evaluate how well the Gaussian approximation captures the true posterior. Detailed results are provided in Supporting Information S3. The VAE achieves an objective function value of −2LL=147682, slightly higher compared to −2LL=147468 obtained by the SAEM, which corresponds to a small difference of less than 0.2%. The reason is that, in this example, the MCMC‐based approximation used by SAEM matches the true posterior $p\left(z|x\right)$ slightly better than the Gaussian like approximation $q_{\psi }\left(z|x\right)$ of the VAE, see Equation (9). Despite this small difference in the approximation of the posterior distribution, both SAEM‐ and VAE‐based methods are well suited to estimate the population parameters.

**FIGURE 4:**
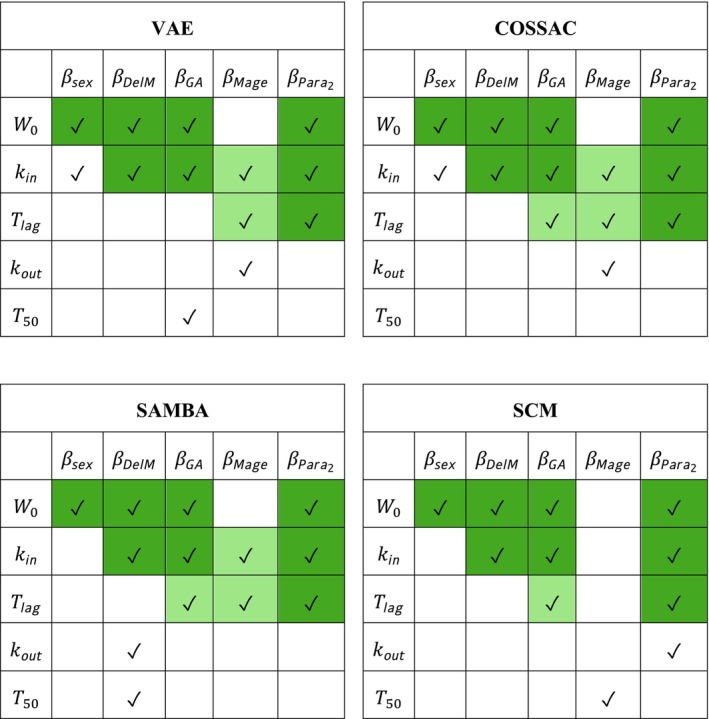
Neonatal weight progression example. Selected covariates for the VAE, COSSAC, SAMBA and SCM models. *Dark green* cells indicate covariates selected by all four methods, while *light green* cells indicate covariates selected by three out of four methods.

**FIGURE 5:**
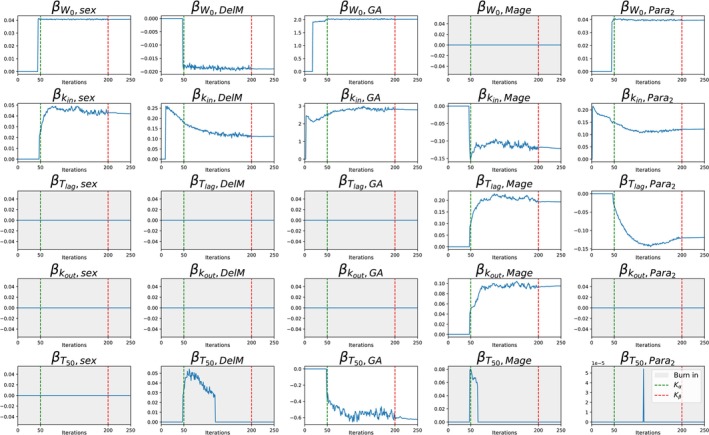
Neonatal weight progression example. Convergence of the covariate effects β for all possible covariate‐parameter combinations. Covariate effects that converge to zero are excluded from the final model are highlighted with a light gray background.

Second, we include covariate selection. The results are presented in Table 2, and the selected covariates are in Figure 4. The convergence of the covariate effects is shown in Figure 5.

**TABLE 2:** Neonatal weight progression example.

Log‐likelihood and BICc values										
	VAE		MCMC		COSSAC		SAMBA		SCM	
	Lin	IS	Lin	IS	Lin	IS	Lin	IS	Lin	IS
−2LL	146370	146406	146120	146154	146086	146132	146121	146177	146116	146123
BICc	146566	146602	146316	146351	146283	146329	146318	146374	**146296**	**146305**
Runs	**1**		**1**		33		2		244	

*Note:* Statistic criteria for the VAE, MCMC with VAE‐fixed covariate model, COSSAC, SAMBA, and SCM. Left: Likelihood computed by linearization method (Lin). Right: Likelihood computed by importance sampling method (IS). Bold values indicate the lowest value.

After covariate selection, the small gap in the log‐likelihood between the VAE and SAEM remains (see Table 2). Compared to its counterparts without covariates, the automatically selected covariates by the VAE in one run shows strong improvement like those of SAMBA, COSSAC or SCM. Combining MCMC posterior with fixed, VAE‐selected covariates closes the gap. SAMBA, COSSAC, and SCM need 2, 33, and 244 runs, respectively, to select similar good covariates. The VAE reaches this result with just one run. The CPU time of this VAE run with covariate selection is 26% longer than without, which is still much faster than two runs (by SAMBA). In summary, the VAE matches the other methods in terms of covariate selection quality, while outperforming them in computational efficiency.

## 4 Discussion

NLME modeling is an essential tool for key decisions in MIDD. However, traditional NLME modeling has its limitations regarding efficacy, especially when it comes to covariate selection, which is an iterative and time‐consuming process.

To address this, we have established VAEs as a powerful AI‐based framework to efficiently solve general NLME modeling problems in PMX. VAEs incorporate the flexibility of AI‐based techniques into solving marginalized likelihood problems (see Equation (4)).

All NLME modeling approaches need approximations of the true posterior distribution $p\left(z|x\right)$. Unlike SAEM's MCMC‐based inference, the VAE approximates the posterior distribution with a smooth Gaussian approximation $q_{\psi }\left(z|x\right)$. This approximation is obtained via a neural network encoder trained directly on the data.

We highlight two aspects of this approximation: (*i*) smoothness and (*ii*) Gaussianity.

- The smooth posterior approximation assumes smooth model structures in the individual parameters. This assumption of smoothness aligns with biological intuition, supports gradient‐based optimization, and enables efficient ELBO convergence. Moreover, future tasks in automated modeling (discussed later) benefit from this smooth structure or even rely on it by design.
- Gaussian approximations make inference faster and computationally cheaper. While SAEM requires repeated ODE solutions for each MCMC sample, the VAE only requires a single encoder pass per sample. Once trained, it can instantly predict posteriors for new individuals. In our experiments, we found that quite often Gaussian approximation closely matched the true posterior, consistent with observations in Janssen et al. [17]. Nevertheless, the VAE framework should ideally be extended in the future to capture non‐Gaussian posterior distributions, potentially improving the accuracy of population parameter estimates in cases where the true posterior is not well approximated by a Gaussian. One possible direction of research are more flexible approximations, such as normalizing flows [30, 31] or mixtures of Gaussians.

The presented VAE‐NLME framework offers a new automated approach to covariate selection by optimizing the adapted information criterion BICc‐ELBO. This enables simultaneous estimation of population parameters and covariate selection in one single run, that is, one run is all you need.

SAMBA, COSSAC, as well as SCM require multiple runs, as they iteratively select a covariate model and compute the corresponding likelihood, testing potential covariate‐parameter relationships. The neonatal weight progression example demonstrates that COSSAC and SCM require a large number of runs, resulting in high computation costs. This is not the case for the VAE. Due to its design and the efficiency of the MIQP solver, the VAE can handle NLME frameworks with hundreds of covariates and parameters. Consequently, the VAE stands out in terms of efficiency and its ability to handle a large number of covariates, while the quality of the selected covariates remains comparable across methods.

Thanks to the design of our VAE‐NLME framework, the model is highly robust. It does not require extensive hyperparameter tuning, and the burn‐in with Kullback–Leibler annealing enables the VAE to consistently find good solutions in our experiments.

A major computational bottleneck in all NLME frameworks is solving ODEs. While optimization is efficient, ODE evaluation dominates runtime. SAEM‐based solutions need more ODE evaluations than other NLME frameworks due to their use of MCMC posterior approximations, compared to Janssen et al. [17]. Hence, faster ODE solvers would benefit all approaches.

The VAE is unlocking the potential of combining AI‐based methods and PMX by offering great flexibility and enabling the integration of advanced AI techniques. Automated covariate selection is one example. Another example could be modification of the encoder to output continuous individual parameters, making it ideal for NLME modeling problems with time‐dependent covariates. Furthermore, certain unknown components of the decoder can be replaced with neural networks, enabling the automated modeling of complex behaviors and offering a more efficient approach to model development. Moreover, VAEs can be applied in PMX to integrate more general data types, such as medical images (e.g., tumor scans in oncology), as well as genomic and metabolomic data [32], as encoder inputs. The VAE paves the way to many other future applications.

In conclusion, the VAE‐NLME framework represents a launch pad into a new era of PMX, where combining AI‐based frameworks and PMX modeling can lead to more accurate and especially more efficient model building and assessment. The presented AI‐PMX approach facilitates automated model development, advancing PMX and its applications in MIDD.

## Author Contributions

J.R., F.B., G.K., and J.S. wrote the manuscript. J.R. and J.S. designed the research. J.R. and J.S. performed the research. J.R., D.B., B.S., U.N., M.P., and G.K. analyzed the data. D.B., B.S., U.N., M.P., and G.K. provided the data. J.R. developed and implemented the complete VAE codebase. All authors reviewed and approved the final manuscript.

## Conflicts of Interest

The authors declare no conflicts of interest.

## Supporting information


**Data S1:** psp470129‐sup‐0001‐Supinfo.zip.
