# Supplementary material for: Redefining Parameter Estimation and Covariate Selection via Variational Autoencoders: One Run Is All You Need
Source: CPT Pharmacometrics Syst Pharmacol. 2025 Nov 4;14(12):2232–43. doi: 10.1002/psp4.70129 (PMC12706427; doi:10.1002/psp4.70129)
Supplement: Supplementary file 1 — Data S1: psp470129‐sup‐0001‐Supinfo.zip. [file PSP4-14-2232-s001.zip › PSP-2025-0205-s01.pdf]

Supplementary Material

A1 Training Parameters for the VAE

All trainings parameters can be found in Table S1 and Table S2.

**Encoder Parameters**	
Latent dimension n_z	3
Hidden dimension n_h	25
Learning rate	$5 \cdot 10^{-3}$
**Initial Values Encoder**	
Individual mean μ_{i,k_a}	1
Individual mean μ_{i,k_e}	0.5
Individual mean $\mu_{i,V}$	15
Individual standard deviation L_i	$\mathrm{diag}(10^{-2}, 10^{-3}, 10^{-1})$
**Decoder Parameters**	
ODE solver	Analytical Solution
**Iteration Parameters**	
Gradient updates per iteration L_{iter}	5
Burn in K_{burn}	100
Kullback-Leibler annealing K_α	50
Smoothing K_γ	250
Iterations K_{iter}	300
Overall iterations K_{total}	300 + 100

Table S1: Theophylline example. Training parameters for the VAE (Case Study 1, Section 3.1).

**Encoder Parameters**	
Latent dimension n_z	5
Hidden dimension n_h	100
Learning rate	$5\cdot 10^{-3}$
**Initial Values Encoder**	
Individual mean μ_{i,W_0}	3000
Individual mean $\mu_{i,k_{in}}$	30
Individual mean $\mu_{i,T_{lag}}$	2
Individual mean $\mu_{i,k_{out}}$	0.05
Individual mean $\mu_{i,T_{50}}$	1
Individual standard deviation L_i	$\mathrm{diag}(10^{-6}, 10^{-4}, 10^{-2}, 10^{-2}, 10^{-2})$
**Decoder Parameters**	
ODE solver	Dopri5
Step size, relative tolerance r_{tol}	10^{-9}
Step size, absolute tolerance a_{tol}	10^{-9}
**Iteration parameters**	
Gradient updates per iteration L_{iter}	10
Burn in K_{burn}	25
Kullback-Leibler annealing K_{α}	50
Smoothing K_{γ}	200
Iterations K_{iter}	250
Overall iterations K_{total}	250 + 25

Table S2: Neonatal weight progression example. Training parameters for the VAE (Case Study 2, Section 3.2)

In conclusion, the VAE requires $K_{total} \cdot L_{iter}$ model evaluations for training. For accurate training via backpropagation, the ODE solver must be sufficiently precise. In this context, extensions to solvers for stiff ODEs, including backward differentiation formula (BDF) methods, are worth mentioning. One of the most effective solvers for such problems is LSODA, which automatically switches between stiff and non-stiff methods.

A2 Robustness of Case Study 1 - Theophylline Pharmacokinetics

To assess the robustness and stability of the VAE, the theophylline example described in the main text in Section 3.1 is solved for different initial random seeds and varying numbers of hidden dimensions $n_h \in \mathbb{N}$ in the LSTM layer. The initial random seed directly affects the initialization of the LSTM weights, as these are sampled from specific distributions: the input-to-hidden weights are initialized from a uniform distribution $\left[-\sqrt{1/n_h}, \sqrt{1/n_h}\right]$ while the hidden-to-hidden weights are initialized orthogonally, and biases are set to zero except for the forget-gate bias. Changing the seed therefore alters the exact starting point of the optimization. Additionally, modifying the number of hidden dimensions n_h changes the architecture of the LSTM, which can affect how the model captures temporal dependencies. This analysis evaluates whether the population parameter estimates θ remain consistent under these variations.

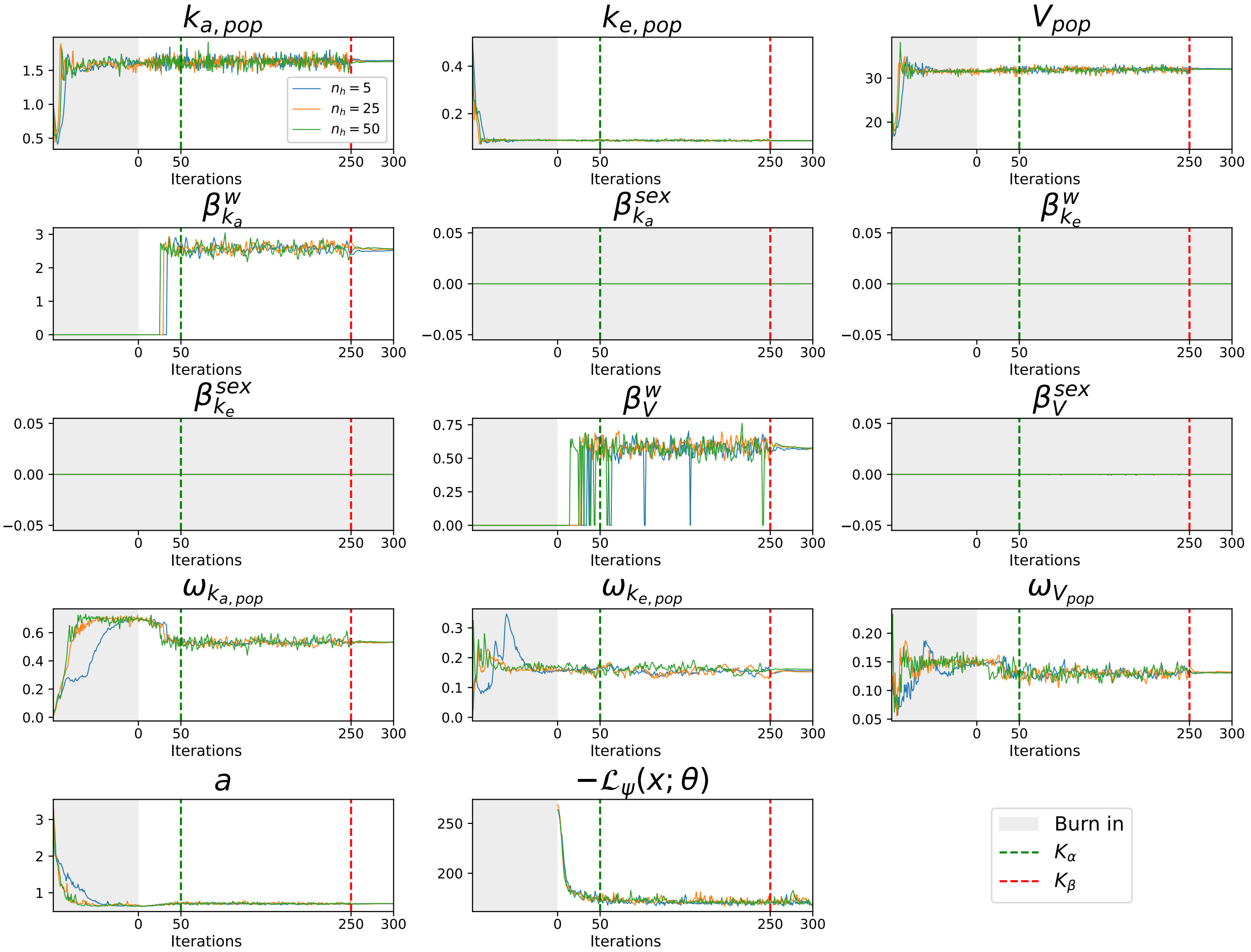

Figure S1: Theophylline example. Convergence of the population parameters θ, including covariate effects β, for the theophylline dataset with three different dimensions $n_h \in \{5,25,50\}$ of the LSTM hidden layer.

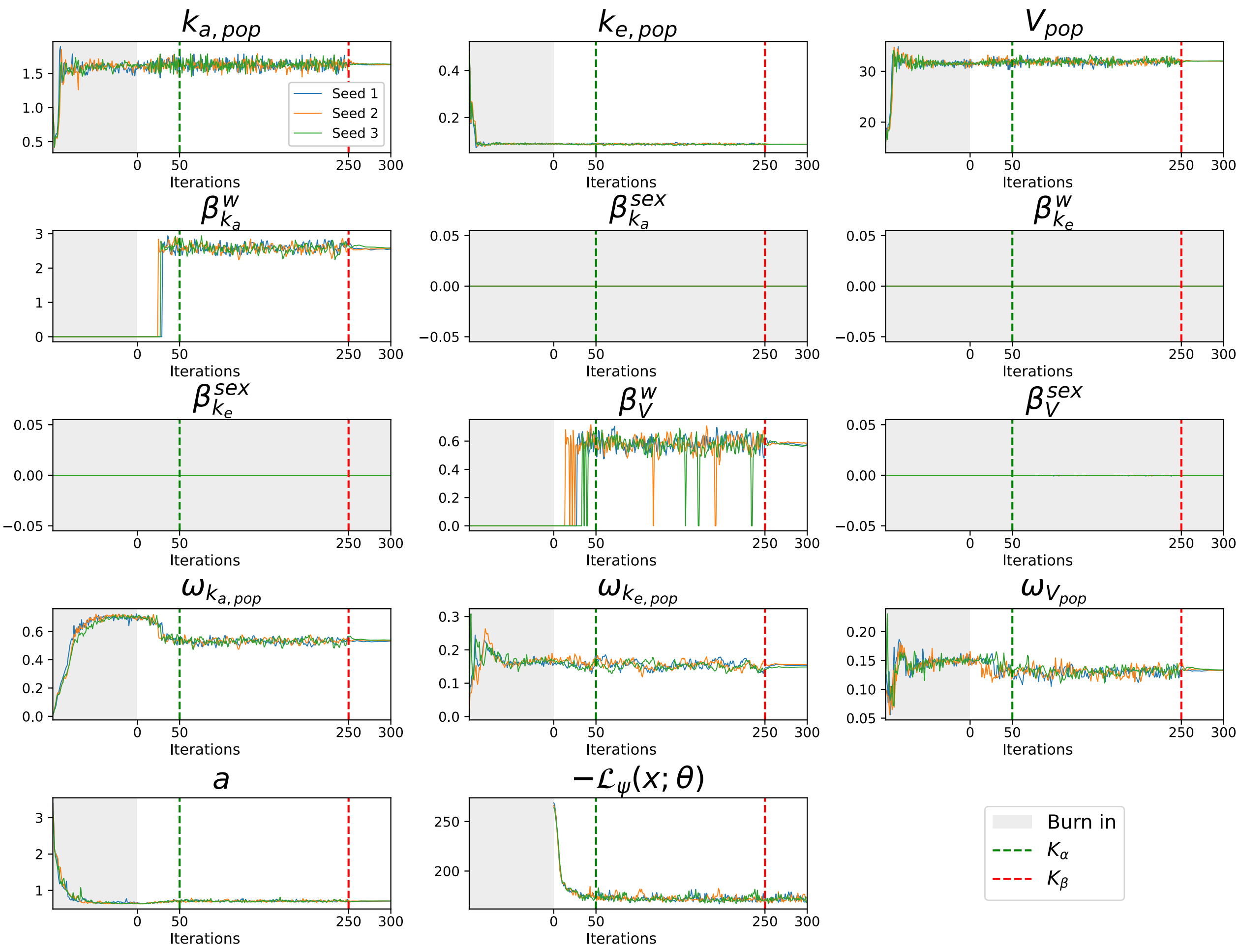

Figure S2: Theophylline example. Convergence of the population parameters θ, including covariate effects β, for the theophylline dataset with three different random seeds.

A3 Population Fit of Case Study 2 - Neonatal Weight Progression

Population parameters from a population fit without any covariates are presented in Table S3.

	VAE		SAEM	
**Fixed Effects**				
$W_{0,pop}$	3383.2		3386.2	
$k_{in,pop}$	106.8		99.6	
$T_{lag,pop}$	1.56		1.61	
$k_{out,pop}$	0.072		0.076	
$T_{50,pop}$	1.18		0.9	
**Standard Deviation**				
ω_{W_0}	0.14		0.14	
$\omega_{k_{in}}$	0.23		0.32	
$\omega_{T_{lag}}$	0.12		0.31	
$\omega_{k_{out}}$	0.10		0.11	
$\omega_{T_{50}}$	0.24		0.14	
**Error Model**				
a	33.0		33.6	
**Stat. Criteria**				
	**Lin**	**IS**	**Lin**	**IS**
$-2\mathcal{LL}$	147681	147682	147360	147468
BICc	147776	147777	147456	147563
$-2\mathcal{LL}/N$	60.90	60.90	60.77	60.82
BICc$/N$	60.94	60.94	60.81	60.85

Table S3: Neonatal weight progression example. Results for the population of the neonate's model (15) for the VAE and MCMC. Statistic criteria are computed by the linearization method (Lin) and by importance sampling method (IS).
