# Supplementary material for: Redefining Parameter Estimation and Covariate Selection via Variational Autoencoders: One Run Is All You Need
Source: CPT Pharmacometrics Syst Pharmacol. 2025 Nov 4;14(12):2232–43. doi: 10.1002/psp4.70129 (PMC12706427; doi:10.1002/psp4.70129)
Supplement: Supplementary file 1 — Data S1: psp470129‐sup‐0001‐Supinfo.zip. [file PSP4-14-2232-s001.zip › PSP-2025-0205-s02.zip!/vae_nlme-code/Plots/theophylline_data.pdf]

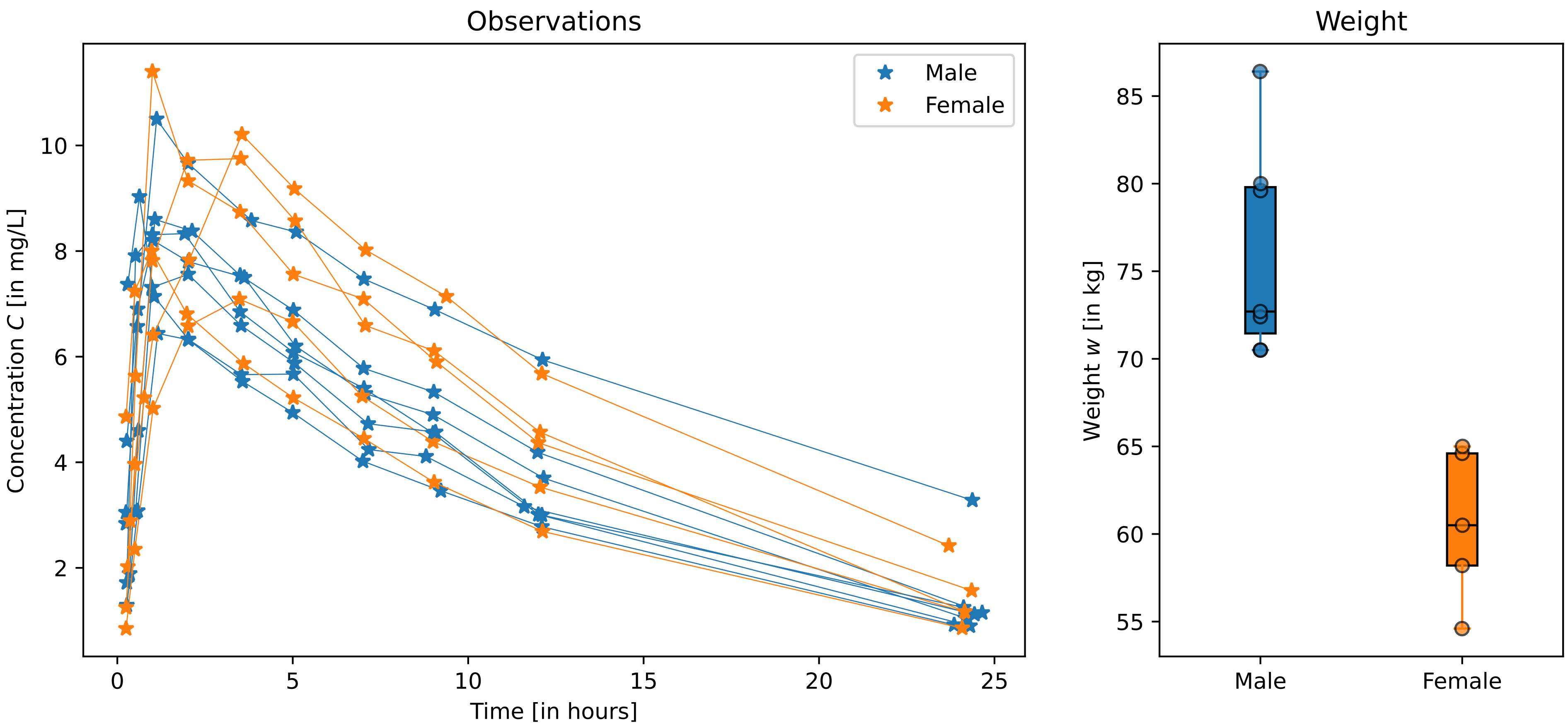
Observations
Male
Female
Concentration C [in mg/L]
Time [in hours]
0
5
10
15
20
25
2
4
6
8
10
Weight
Weight w [in kg]
55
60
65
70
75
80
85
Male
Female
