# Supplementary material for: Redefining Parameter Estimation and Covariate Selection via Variational Autoencoders: One Run Is All You Need
Source: CPT Pharmacometrics Syst Pharmacol. 2025 Nov 4;14(12):2232–43. doi: 10.1002/psp4.70129 (PMC12706427; doi:10.1002/psp4.70129)
Supplement: Supplementary file 1 — Data S1: psp470129‐sup‐0001‐Supinfo.zip. [file PSP4-14-2232-s001.zip › PSP-2025-0205-s02.zip!/vae_nlme-code/Plots/theophylline_convergence_popParam.pdf]

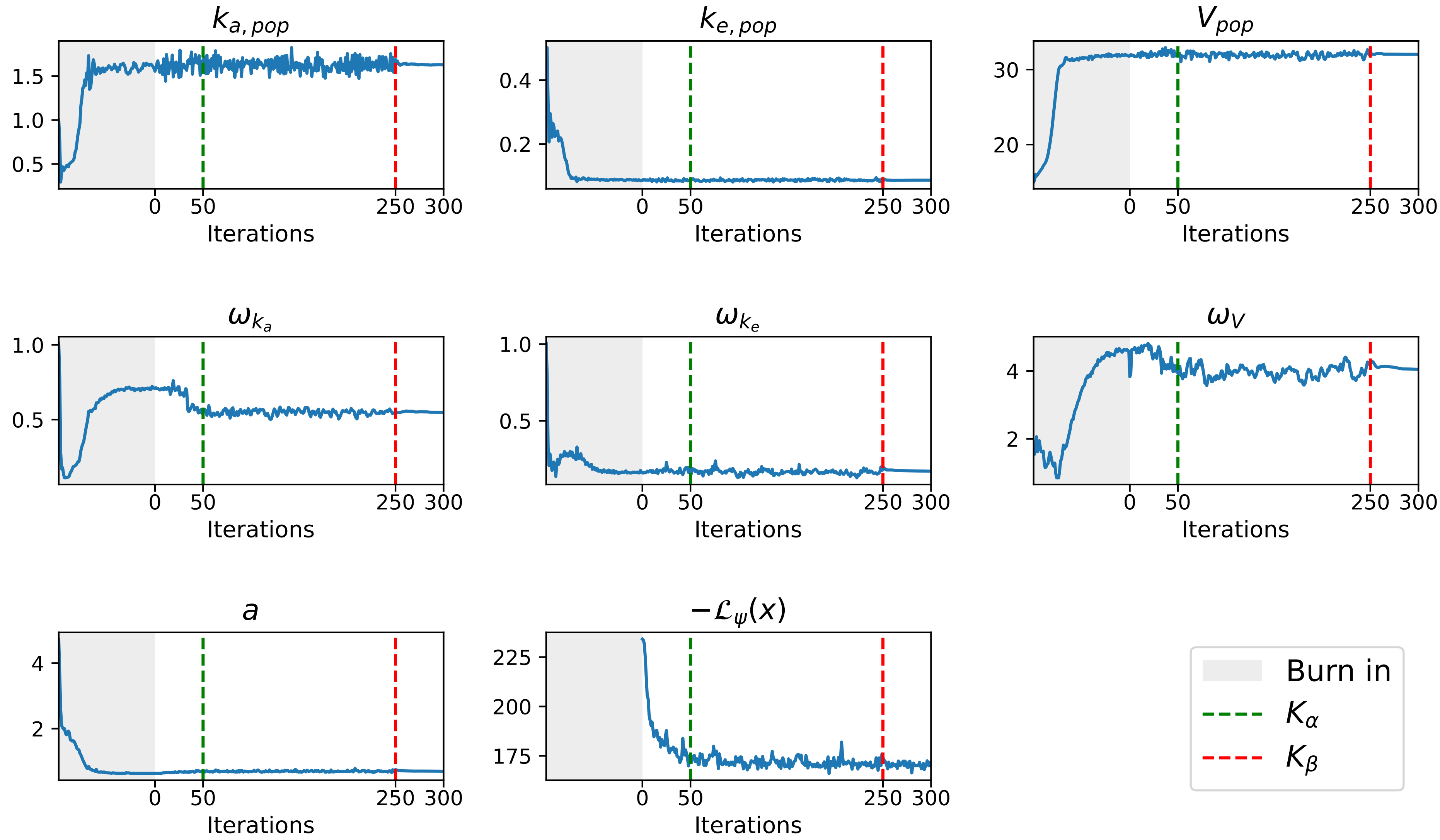

$k_{a,pop}$
$k_{e,pop}$
V_{pop}
ω_{k_a}
ω_{k_e}
ω_V
a
$-\mathcal{L}_\psi(x)$
Iterations
0
50
250
300
1.5
1.0
0.5
0.4
0.2
30
20
4
2
225
200
175
Burn in
K_α
K_β
