# Supplementary material for: Redefining Parameter Estimation and Covariate Selection via Variational Autoencoders: One Run Is All You Need
Source: CPT Pharmacometrics Syst Pharmacol. 2025 Nov 4;14(12):2232–43. doi: 10.1002/psp4.70129 (PMC12706427; doi:10.1002/psp4.70129)
Supplement: Supplementary file 1 — Data S1: psp470129‐sup‐0001‐Supinfo.zip. [file PSP4-14-2232-s001.zip › PSP-2025-0205-s02.zip!/vae_nlme-code/Plots/neonates_convergence_covariate.pdf]

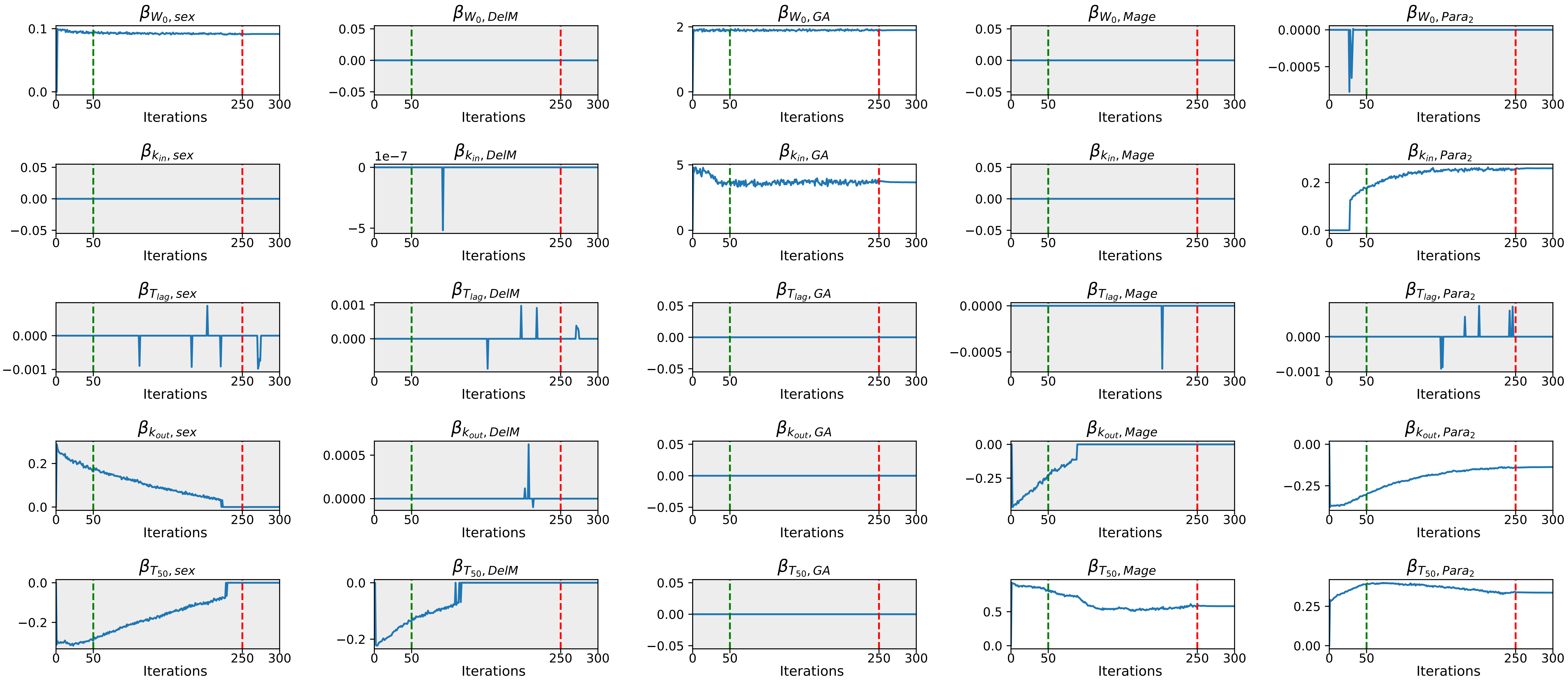

$\beta_{W_0, sex}$
0.1
0.0
$\beta_{W_0, DelM}$
0.05
0.00
−0.05
$\beta_{W_0, GA}$
2
0
$\beta_{W_0, Mage}$
0.05
0.00
−0.05
$\beta_{W_0, Para_2}$
0.0000
−0.0005
$\beta_{k_{in}, sex}$
0.05
0.00
−0.05
$\beta_{k_{in}, DelM}$
1e−7
0
−5
$\beta_{k_{in}, GA}$
5
0
$\beta_{k_{in}, Mage}$
0.05
0.00
−0.05
$\beta_{k_{in}, Para_2}$
0.2
0.0
$\beta_{T_{lag}, sex}$
0.000
−0.001
$\beta_{T_{lag}, DelM}$
0.001
0.000
$\beta_{T_{lag}, GA}$
0.05
0.00
−0.05
$\beta_{T_{lag}, Mage}$
0.0000
−0.0005
$\beta_{T_{lag}, Para_2}$
0.000
−0.001
$\beta_{k_{out}, sex}$
0.2
0.0
$\beta_{k_{out}, DelM}$
0.0005
0.0000
$\beta_{k_{out}, GA}$
0.05
0.00
−0.05
$\beta_{k_{out}, Mage}$
0.00
−0.25
$\beta_{k_{out}, Para_2}$
0.00
−0.25
$\beta_{T_{50}, sex}$
0.0
−0.2
$\beta_{T_{50}, DelM}$
0.0
−0.2
$\beta_{T_{50}, GA}$
0.05
0.00
−0.05
$\beta_{T_{50}, Mage}$
0.5
0.0
$\beta_{T_{50}, Para_2}$
0.25
0.00
0 50 250 300
Iterations
