# Supplementary material for: Redefining Parameter Estimation and Covariate Selection via Variational Autoencoders: One Run Is All You Need
Source: CPT Pharmacometrics Syst Pharmacol. 2025 Nov 4;14(12):2232–43. doi: 10.1002/psp4.70129 (PMC12706427; doi:10.1002/psp4.70129)
Supplement: Supplementary file 1 — Data S1: psp470129‐sup‐0001‐Supinfo.zip. [file PSP4-14-2232-s001.zip › PSP-2025-0205-s02.zip!/vae_nlme-code/Plots/theophylline_convergence_covariate.pdf]

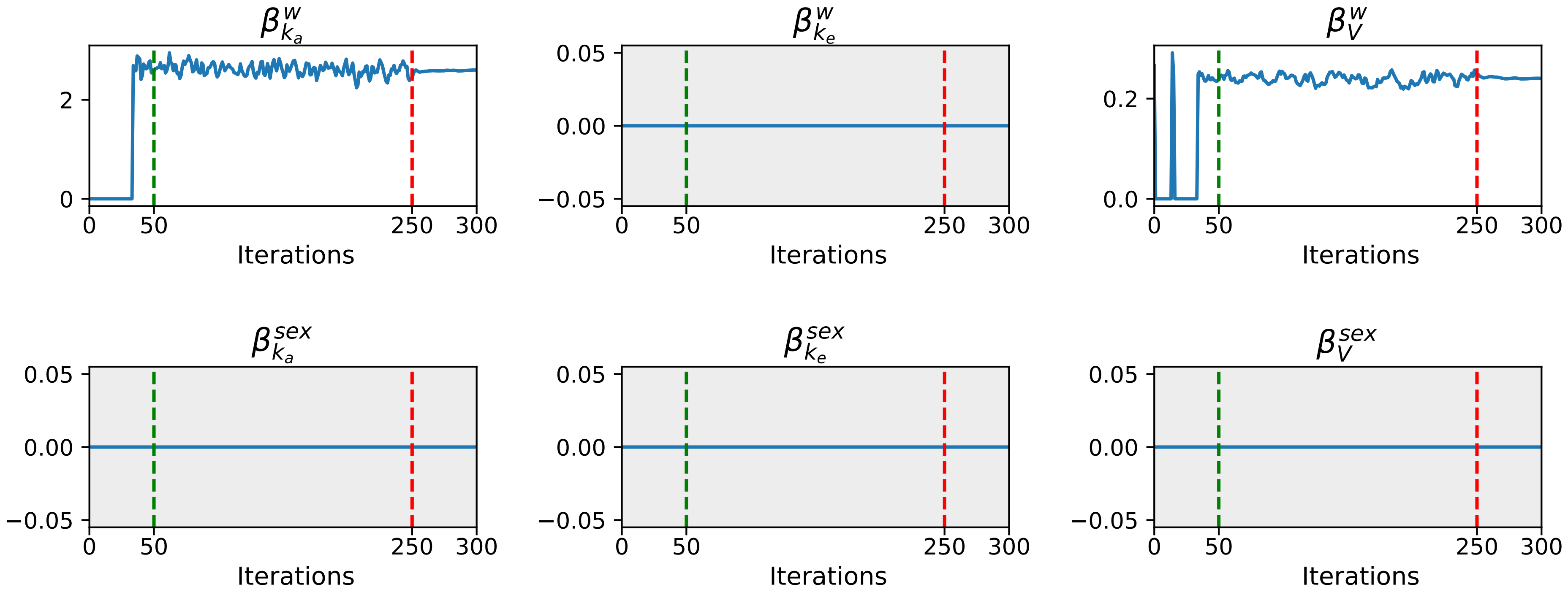
$\beta^{w}_{k_a}$
$\beta^{w}_{k_e}$
β^{w}_{V}
$\beta^{sex}_{k_a}$
$\beta^{sex}_{k_e}$
β^{sex}_{V}
Iterations
0
2
0.05
0.00
−0.05
0.0
0.2
0 50 250 300
