# Supplementary material for: Redefining Parameter Estimation and Covariate Selection via Variational Autoencoders: One Run Is All You Need
Source: CPT Pharmacometrics Syst Pharmacol. 2025 Nov 4;14(12):2232–43. doi: 10.1002/psp4.70129 (PMC12706427; doi:10.1002/psp4.70129)
Supplement: Supplementary file 1 — Data S1: psp470129‐sup‐0001‐Supinfo.zip. [file PSP4-14-2232-s001.zip › PSP-2025-0205-s02.zip!/vae_nlme-code/Plots/neonates_convergence_popParam.pdf]

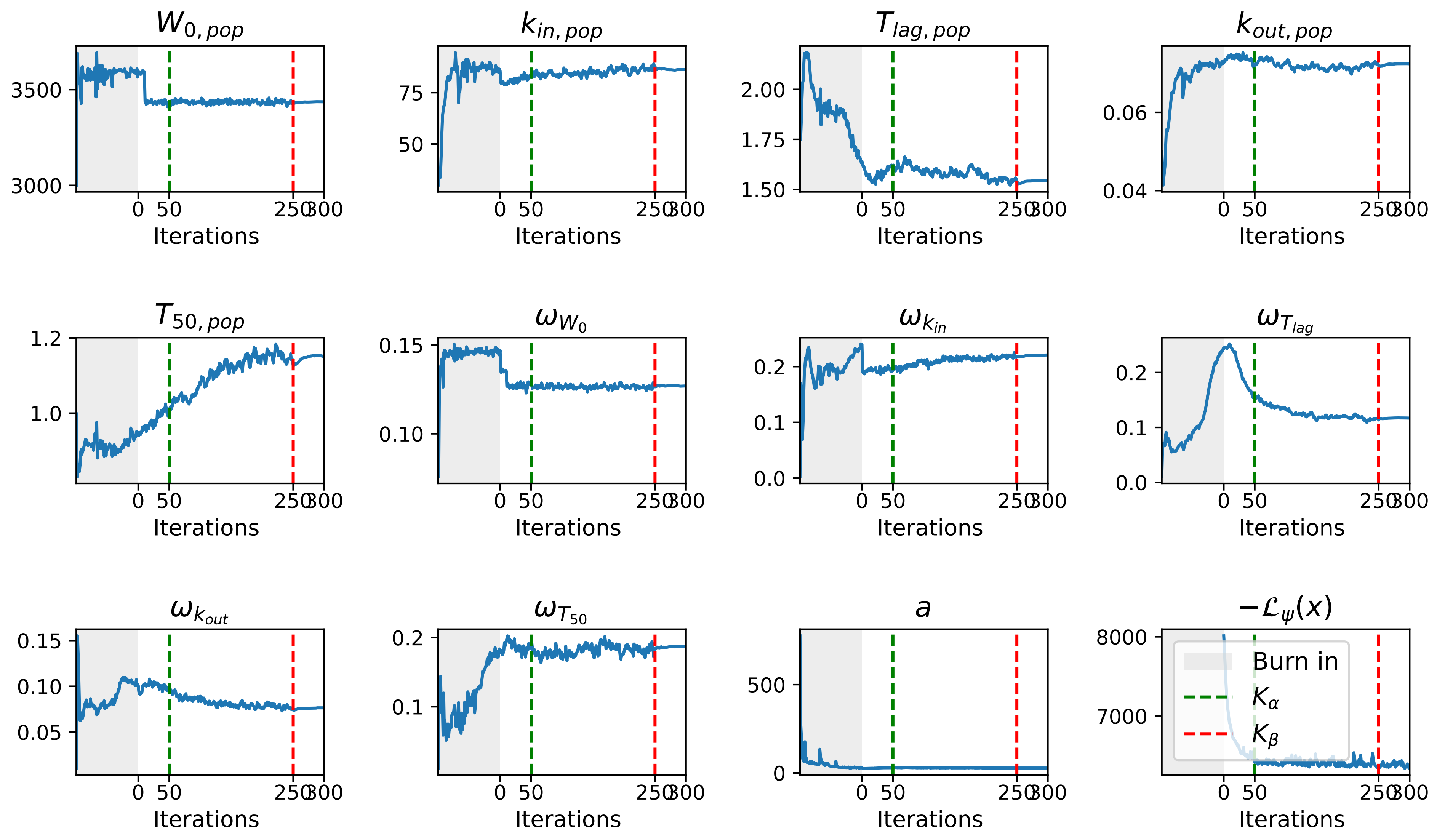

$W_{0,pop}$
$k_{in,pop}$
$T_{lag,pop}$
$k_{out,pop}$
$T_{50,pop}$
ω_{W_0}
$\omega_{k_{in}}$
$\omega_{T_{lag}}$
$\omega_{k_{out}}$
$\omega_{T_{50}}$
a
$-\mathcal{L}_\psi(x)$
Iterations
Burn in
K_α
K_β
