# Supplementary material for: Redefining Parameter Estimation and Covariate Selection via Variational Autoencoders: One Run Is All You Need
Source: CPT Pharmacometrics Syst Pharmacol. 2025 Nov 4;14(12):2232–43. doi: 10.1002/psp4.70129 (PMC12706427; doi:10.1002/psp4.70129)
Supplement: Supplementary file 1 — Data S1: psp470129‐sup‐0001‐Supinfo.zip. [file PSP4-14-2232-s001.zip › PSP-2025-0205-s02.zip!/vae_nlme-code/Plots/neonates_data.pdf]

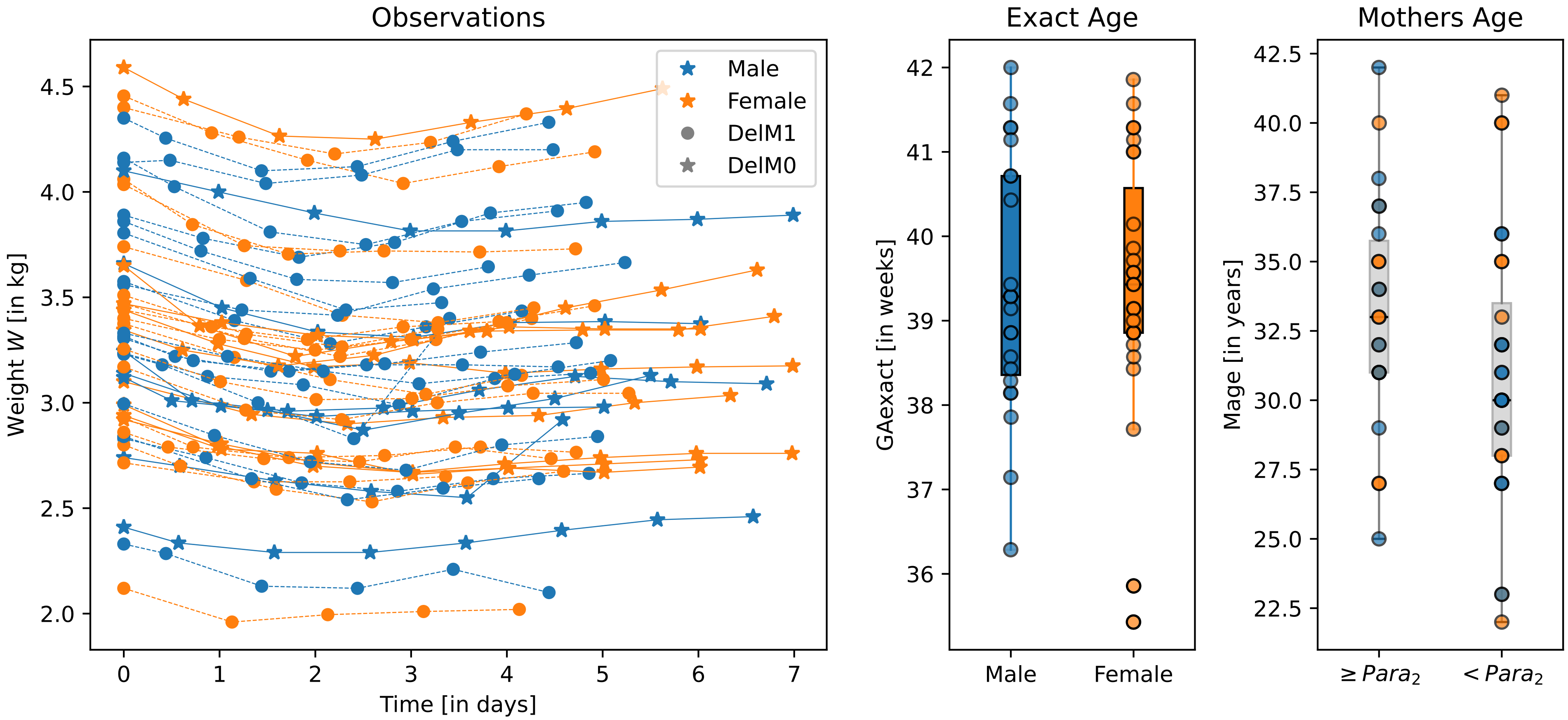
Observations
Male
Female
DelM1
DelM0
Weight W [in kg]
Time [in days]
4.5
4.0
3.5
3.0
2.5
2.0
0
1
2
3
4
5
6
7
Exact Age
GAexact [in weeks]
42
41
40
39
38
37
36
Male
Female
Mothers Age
Mage [in years]
42.5
40.0
37.5
35.0
32.5
30.0
27.5
25.0
22.5
≥ Para2
< Para2
